# Clinical efficacy and cortical network modulation of a multimodal sensory feedback upper-limb rehabilitation robot for post-stroke upper-limb recovery: a randomized controlled trial

**DOI:** 10.3389/fneur.2026.1820579

**Published:** 2026-09-04

**Authors:** Li-Hui Zhao, Jun-Peng Zhang, Jia-Jia Wu, Yu Cao, Zhan-Yue Lu, Xu-Yun Hua, Xia Bi, Jian-Guang Xu

**Affiliations:** 1 School of Rehabilitation Science, Shanghai University of Traditional Chinese Medicine, Shanghai, China; 2 Engineering Research Center of Traditional Chinese Medicine Intelligent Rehabilitation, Ministry of Education, Shanghai, China; 3 Department of Rehabilitation Medicine,Yueyang Hospital of Integrated Traditional Chinese Medicine, Shanghai, China; 4 Department of Rehabilitation Medicine, Zhoupu Hospital, Shanghai, China; 5 Department of Traumatology and Orthopedics, Shuguang Hospital Affiliated to Shanghai University of Traditional Chinese Medicine, Shanghai, China

**Keywords:** cortical activation, fNIRS, functional connectivity, multimodal sensory feedback, neuroplasticity, robot-assisted therapy, stroke, upper-limb rehabilitation

## Abstract

**Background:**

Recovery of upper-limb motor function after stroke often requires intensive, repetitive, task-oriented rehabilitation. Multimodal sensory-feedback robot-assisted training has the potential to enhance patient engagement and sensorimotor integration, and thereby improve rehabilitation outcomes.

**Aim:**

To investigate the clinical and neurophysiological effects of adding multimodal sensory-feedback robot-assisted training to conventional rehabilitation for post-stroke upper-limb recovery using functional near-infrared spectroscopy (fNIRS).

**Methods:**

A total of 38 stroke patients were randomly assigned to a control group (conventional rehabilitation, *n* = 19) or an experimental group (dose-matched conventional rehabilitation plus multimodal sensory-feedback robot-assisted training, *n* = 19). Upper-limb motor function was assessed before and after intervention using the Fugl-Meyer Assessment for the Upper Extremity (FMA-UE). fNIRS was used to evaluate resting-state functional connectivity (Fisher z-transformed connectivity, zFC) and task-evoked cortical activation (HbO) during shoulder, elbow, and finger flexion tasks.

**Results:**

No significant between-group difference in FMA-UE was observed at baseline (*p* > 0.05). After intervention, the experimental group showed significant improvement in FMA-UE, whereas the control group showed a smaller, non-significant improvement; the magnitude of pre–post change differed significantly between groups (*p* < 0.05). Resting-state fNIRS showed no significant between-group differences in zFC at baseline, whereas post-intervention analyses showed enhanced fronto-motor zFC in the experimental group, including connections involving PreM&SMC, DLPFC, and FEF. Task-fNIRS revealed channel-specific group × time interaction effects in HbO responses during the three motor tasks; *post-hoc* analyses showed significant post-intervention reductions in cortical activation at selected channels in the control group, whereas the experimental group showed a relatively preserved activation pattern.

**Conclusion:**

Adding multimodal sensory-feedback robot-assisted training to dose-matched conventional rehabilitation may improve short-term post-stroke upper-limb motor recovery. fNIRS further revealed intervention-related changes in cortical activation and resting-state functional connectivity.

**Clinical trial registration:**

Identifier, ChiCTR2400080101.

## 1 Introduction

Stroke-related upper-limb motor impairment remains highly prevalent and disabling, with more than half of survivors experiencing persistent deficits that compromise activities of daily living and quality of life (1). Conventional rehabilitation improves outcomes, yet the achievable training dose is often constrained by therapist time, patient fatigue, and limited opportunities for high-repetition task practice (2). These barriers are particularly relevant for upper-limb recovery, where intensive, task-oriented, and feedback-rich practice is a key driver of experience-dependent neuroplasticity (3).

Robot-assisted upper-limb rehabilitation offers a scalable approach to deliver high-intensity, repeatable, and standardized training beyond what is typically feasible with conventional therapy alone (4). Meta-analytic evidence supports robotic training for improving upper-limb motor outcomes after stroke (5), and recent development has increasingly emphasized human–robot interaction, compliant control, and adaptive algorithms to optimize benefit across patient subgroups (6). In parallel, virtual reality (VR) and multimodal feedback technologies have evolved from primarily visual feedback toward more immersive multisensory environments, including auditory cues, virtual rewards, and haptic/force-related feedback (7). Such multimodal sensory feedback is theorized to enhance rehabilitation by strengthening sensorimotor integration (8), promoting attention and engagement (9), and supporting error-based motor learning (10, 11)—mechanisms that collectively facilitate synaptic plasticity and network reorganization (12, 13).

Despite these advances, clinical evidence remains limited regarding whether adding multimodal sensory feedback robot-assisted training to dose-matched conventional rehabilitation yields superior short-term functional gains, and whether such gains are accompanied by measurable changes in cortical activation and connectivity. Functional near-infrared spectroscopy (fNIRS) provides a practical, non-invasive method to monitor cortical hemodynamics with relatively high tolerance to movement (14) and is increasingly used to probe neurophysiological correlates of rehabilitation (15).

Therefore, we conducted a randomized controlled trial to evaluate the efficacy of a multimodal sensory-feedback upper-limb rehabilitation robotic system as an adjunct to conventional rehabilitation in patients after stroke. We hypothesized that, compared with dose-matched conventional rehabilitation alone, robot-assisted training would (i) produce greater improvement in upper-limb motor function, and (ii) enhance cortical activation and functional connectivity measured by fNIRS during resting-state and task-state paradigms.

## 2 Methods

### 2.1 Participants sample size estimation

This single-center randomized controlled trial enrolled patients with stroke who were treated at the Department of Rehabilitation Medicine, Zhoupu Hospital, Pudong New Area, Shanghai, between January 2024 and October 2025. The diagnosis of stroke (ischemic or hemorrhagic) was established according to the 2019 Chinese key diagnostic criteria for major cerebrovascular diseases and confirmed by CT or MRI.

Inclusion criteria were: (1) confirmed stroke (ischemic/hemorrhagic); (2) age 40–75 years; (3) time since stroke 2 weeks to 1 year; (4) right-handed; (5) sitting balance grade ≥II; (6) Modified Ashworth Scale score <2 in the affected upper limb; and (7) ability to understand the procedures and provide written informed consent.

Exclusion criteria were: (1) clinical deterioration (e.g., new infarction/hemorrhage); (2) history of epilepsy, major psychiatric disorders, or severe cognitive/emotional dysfunction precluding cooperation; (3) orthopedic or other conditions limiting limb movement (e.g., fracture, severe arthritis, amputation); (4) history of medications known to substantially affect central nervous system function that could confound outcomes; and (5) pregnancy or lactation.

### 2.2 Sample size estimation

Sample size was calculated for comparing mean changes between two independent groups (two-sided α = 0.05; power 80%, β = 0.20). Based on pilot observations, the expected between-group difference in change of Fugl–Meyer Assessment—Upper Extremity (FMA-UE) score was δ = 2.27 points, with standard deviation σ = 2.28, and equal allocation (K = 1). Using:

$$n1=\frac{K+1}{K}\times \frac{{\left(Z_{\alpha }+Z_{\beta }\right)}^{2}\sigma^{2}}{\delta^{2}}$$

The minimum required sample size was 16 participants per group. Allowing for 15% drop-out, the final sample size was 38 participants.

### 2.3 Randomization and allocation concealment

**Figure 1:**
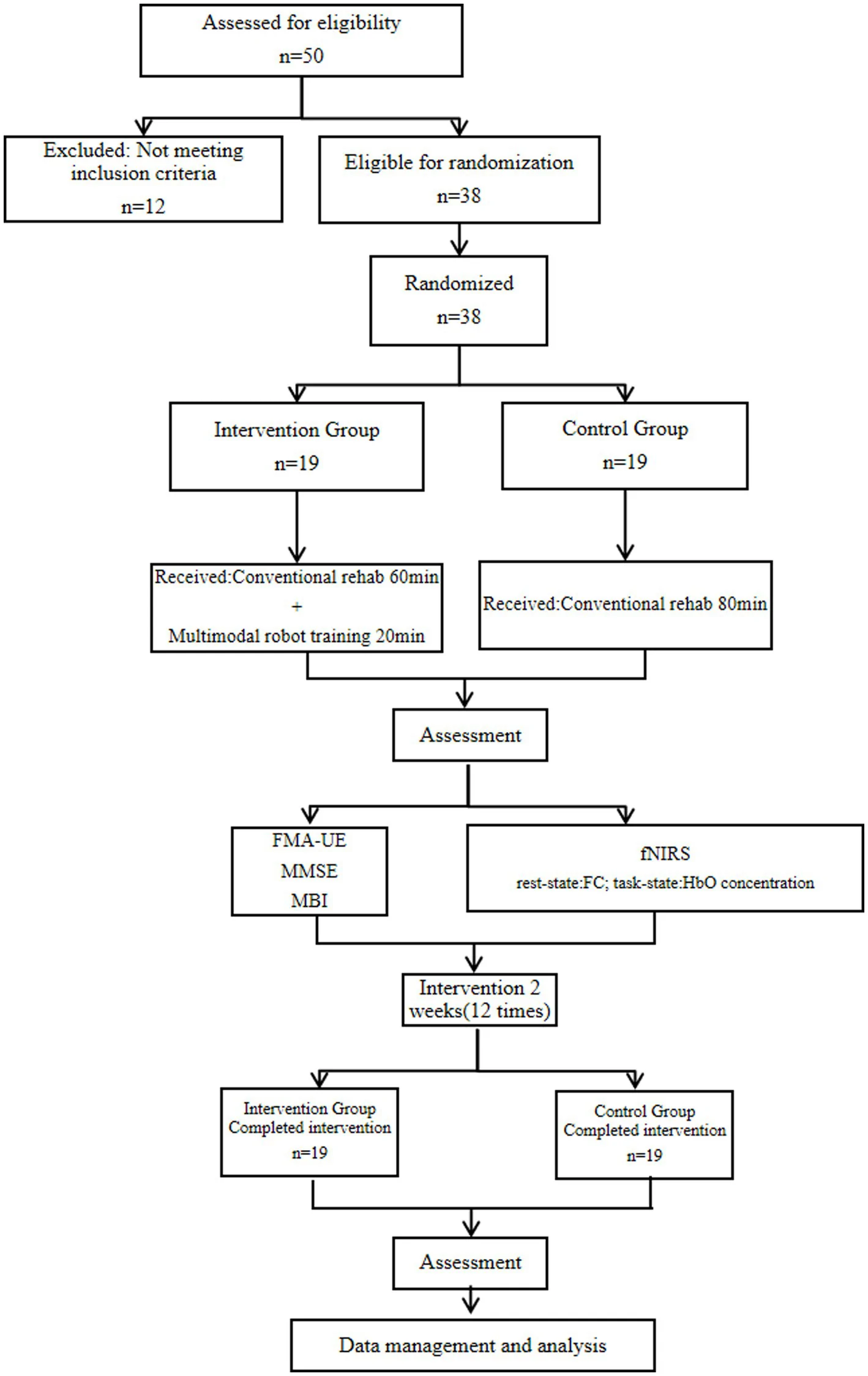
Flowchart through this study.

Participants were randomly assigned (1:1) to the control group or experimental group using a random number table generated in SPSS (version 25.0). Allocation was concealed using sequentially numbered, opaque, sealed envelopes containing group assignment cards prepared by personnel not involved in enrollment or outcome assessment. Participants were allocated according to the order of enrollment. The CONSORT flow diagram is presented in Figure 1.

### 2.4 Blinding

Because of the nature of the intervention, participants and treating therapists were not blinded. Outcome assessments were conducted by the same occupational therapist who was blinded to group allocation. Participants were instructed not to disclose their intervention to the assessor.

### 2.5 Trial design and interventions

Both groups received conventional rehabilitation delivered by licensed rehabilitation therapists, 60 min/day, 6 days/week, for 2 weeks. Conventional rehabilitation comprised:

(a) Physical therapy(30 min): (i) passive and active-assisted range-of-motion (ROM) exercises for the affected upper-limb joints (shoulder, elbow, and wrist) in all available directions; (ii) progressive strengthening exercises for the proximal upper-limb muscles using elastic resistance; and (iii) trunk stability and postural control training with weight-bearing through the affected upper limb. Exercise intensity was progressively increased according to predefined criteria. Advancement to the next difficulty level was permitted only when participants were able to complete three sets of 12 repetitions with appropriate movement quality and without excessive compensatory movements.
(b) Occupational therapy (20 min): occupational therapy consisted of graded task-oriented training individualized according to baseline upper-limb motor impairment assessed by the Fugl–Meyer Assessment of the Upper Extremity (FMA-UE). Participants with severe impairment (FMA-UE 18–30) practiced basic reaching and bilateral coordination tasks, including towel sliding, large-block stacking, and bilateral compression activities. Participants with moderate impairment (FMA-UE 31–45) performed functional reaching, grasping, and object-manipulation tasks, including turning large screws, simulated door opening, and unilateral cup grasping. Participants with mild-to-moderate impairment (FMA-UE ≥ 46) received more challenging dexterity training involving bead threading, tweezer manipulation, and simulated feeding activities to improve fine motor function and activities of daily living.
(c) Patient education (10 min): stroke-related education addressing secondary prevention, home exercise, and daily-life precautions was provided to both patients and caregivers.

To ensure equivalent treatment dosage between groups, participants in the control group received an additional 20 min of conventional rehabilitation, resulting in identical total treatment time across groups.

In addition to conventional rehabilitation, participants in the experimental group received robot-assisted upper-limb training using the ArmGuider® system (Shanghai Zhuodao Medical Technology Co., Ltd., Shanghai, China) for 20 min/session, 1 session/day, 6 days/week, for 2 consecutive weeks.

The ArmGuider® system is a planar upper-limb rehabilitation robotic device based on a parallel five-bar mechanism, designed to provide two-dimensional trajectory-guided training with real-time motion multimodal sensory feedback. In the present study, multimodal sensory feedback was operationally defined as the simultaneous provision of visual, auditory, proprioceptive, and tactile feedback within a closed-loop human–robot interaction system. Visual feedback was provided through an interactive graphical interface displaying movement trajectories, target positions, and task performance to facilitate motor planning and online error correction. Auditory feedback consisted of verbal instructions and task-related performance cues that reinforced successful task completion and maintained participant engagement. Proprioceptive feedback was generated through robot-assisted limb movements, enabling participants to continuously perceive joint position, movement trajectory, and limb displacement during training. Tactile feedback arose from the physical interaction between the robotic end-effector and the participant’s upper limb, providing continuous sensory information regarding movement guidance and contact forces. Rather than functioning independently, these feedback modalities were delivered simultaneously throughout training to enhance sensorimotor integration, reinforce movement-related sensory information, and facilitate motor relearning.

**Figure 2:**
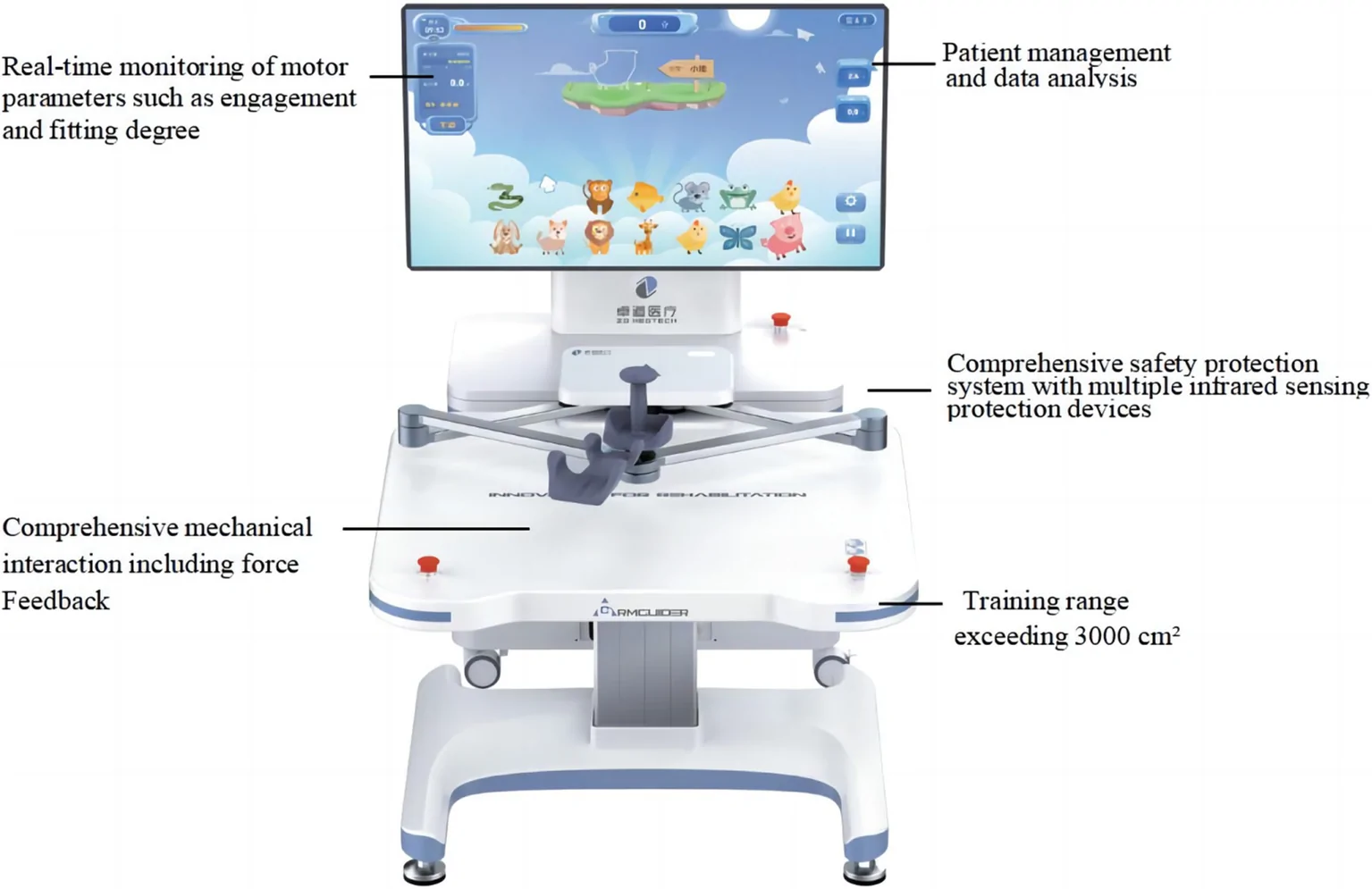
ArmGuider® multimodal sensory-feedback upper-limb rehabilitation robot system used in the experimental intervention. Annotated system overview showing the planar training mechanism, interactive display interface, force-feedback interaction module, infrared safety protection components, and patient management/data analysis functions.

**Figure 3:**
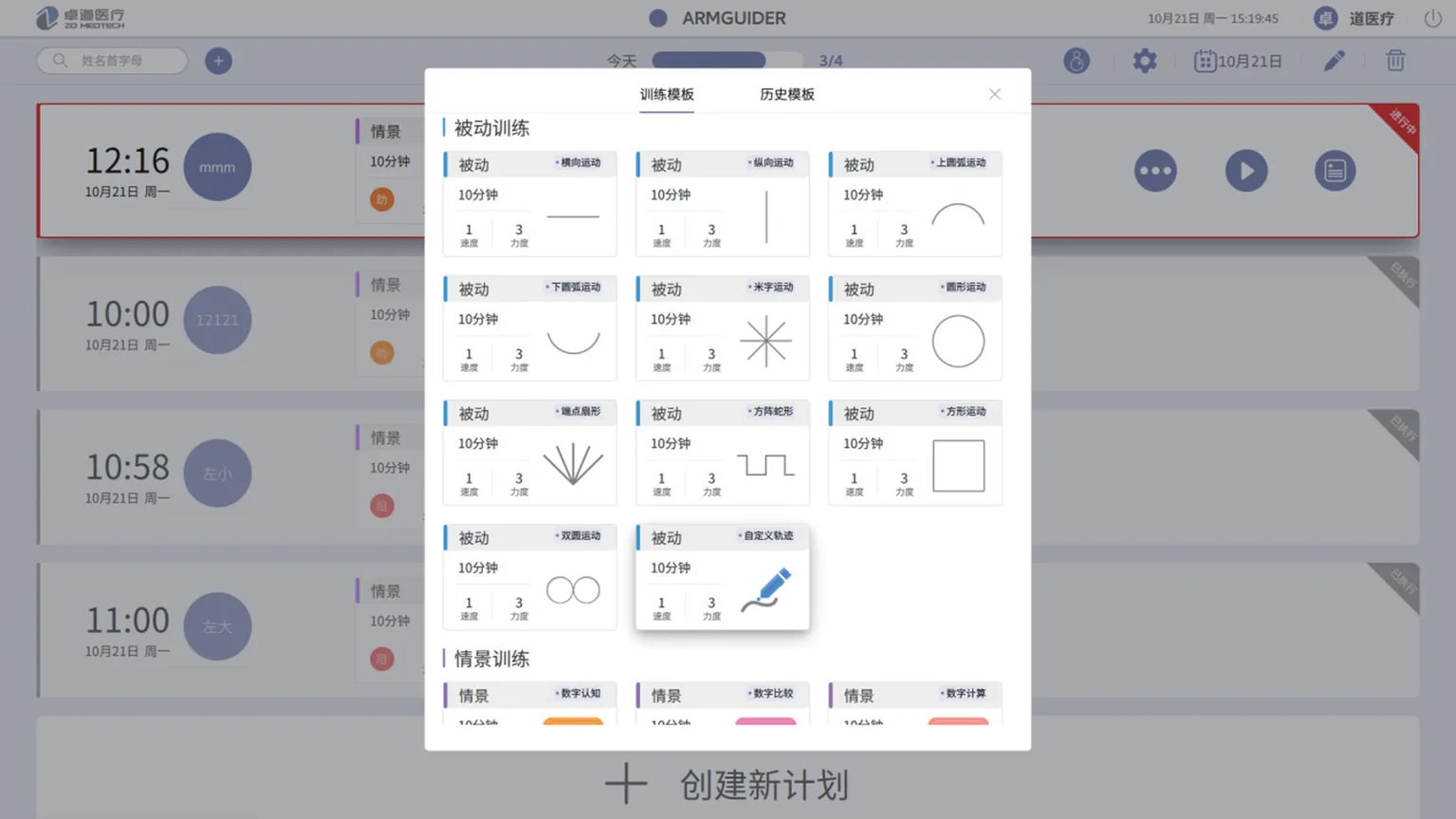
Passive training interface of the ArmGuider® multimodal sensory-feedback upper-limb rehabilitation robot system. Representative screenshot of the passive training module showing selectable passive trajectory templates and adjustable training parameters (e.g., duration, speed, and assistance/intensity settings) used in the experimental intervention.

**Figure 4:**
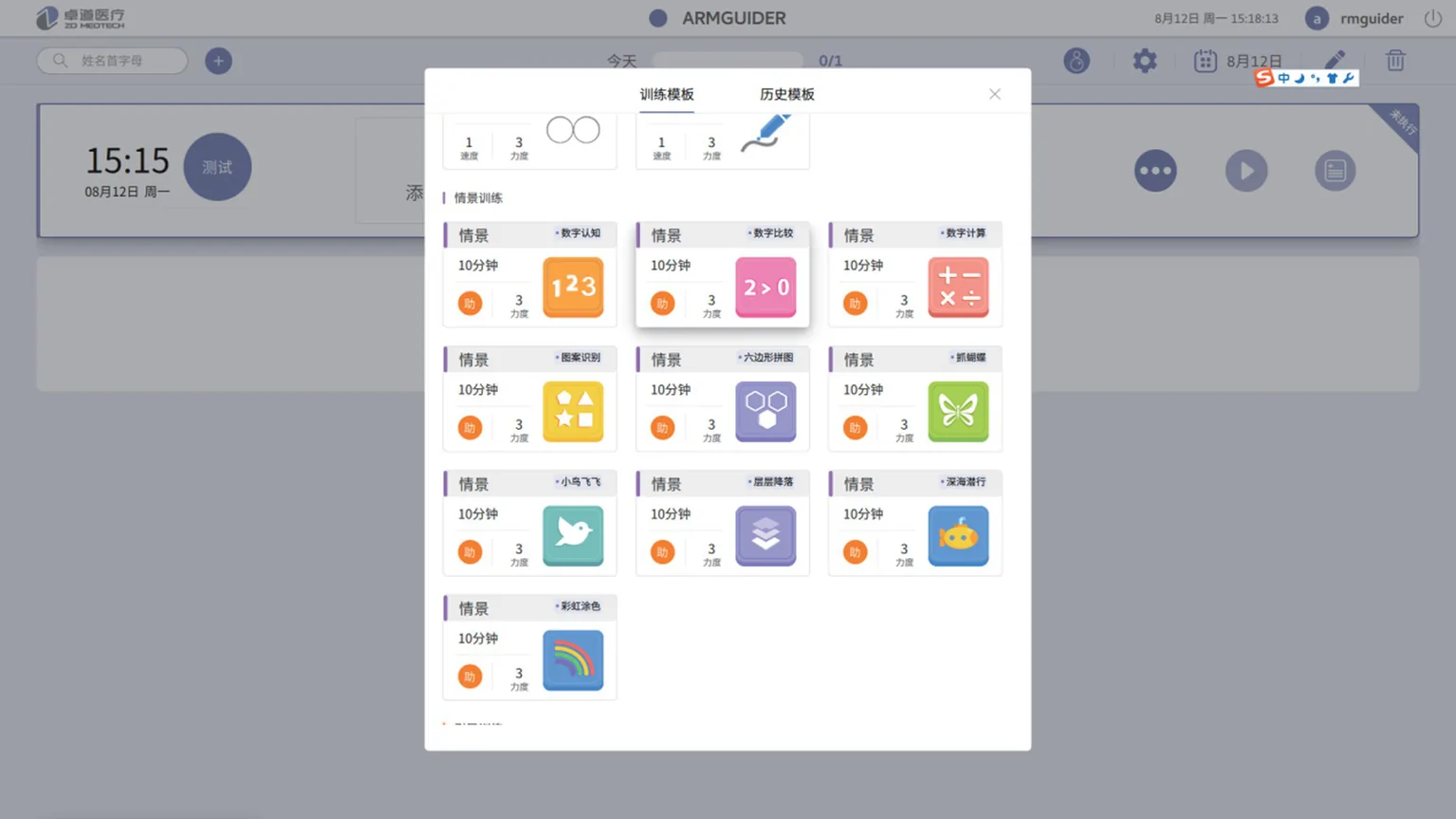
Scenario-based training interface of the ArmGuider® multimodal sensory-feedback upper-limb rehabilitation robot system. The screenshot shows the task-selection module used for scenario-based training, including multiple gamified task templates, adjustable training parameters (e.g., duration and assistance level), and interactive visual feedback options.

The system supports multiple training modes, including passive training, guided/assistive training, and task-oriented scenario-based training with real-time performance feedback (Figure 2). Each training session consisted of 10 min of passive training followed by 10 min of scenario-based training (Figures 3, 4). The first 10 min consisted of passive trajectory-guided training, during which the robotic system guided the affected upper limb through predefined movement trajectories to maintain joint mobility and provide repeated proprioceptive input. The subsequent 10 min consisted of task-oriented interactive training in which participants actively performed functional movement tasks with adaptive robotic assistance and continuous multimodal sensory feedback.

Training parameters, including movement speed, robotic assistance, trajectory complexity, and task difficulty, were individualized according to each participant’s baseline motor impairment and active movement capacity. Participants with severe impairment (FMA-UE 18–30) received higher levels of robotic assistance and practiced simple single-joint or proximal reaching movements to facilitate movement initiation. Participants with moderate impairment (FMA-UE 31–45) performed multi-joint reaching tasks with moderate robotic assistance designed to improve inter-joint coordination and movement accuracy. Participants with mild-to-moderate impairment (FMA-UE ≥ 46) received minimal robotic assistance or resistance-based training while performing multidirectional functional reaching and manipulation tasks intended to improve movement precision, muscular endurance, and motor control.

All robotic training sessions were delivered individually by therapists with more than 5 years of neurological rehabilitation experience who had completed standardized training in operation of the robotic system before participant recruitment. Robotic assistance was progressively reduced as motor performance improved according to the same predefined progression criteria used during conventional rehabilitation. Throughout the intervention, therapists monitored movement quality and participant safety, adjusting assistance only within the predefined treatment algorithm.

Training adherence and intervention fidelity were monitored throughout the study. The robotic system automatically recorded training duration, task completion, movement trajectories, robotic assistance levels, and participant active contribution for every session. These records were reviewed regularly to ensure protocol adherence and treatment consistency across participants.

To ensure participant safety, therapists continuously monitored pain, fatigue, blood pressure, and overall tolerance during training. Robotic training was immediately interrupted if participants experienced excessive pain (visual analog scale score >4), marked hypertension (systolic blood pressure >180 mmHg or diastolic blood pressure >110 mmHg), dizziness, or any other adverse symptoms.

### 2.6 Outcome measures

Assessments were performed at baseline (pre-intervention) and immediately after the 2-week intervention.

The primary outcome was the change in Fugl–Meyer Assessment–Upper Extremity (FMA-UE) score from baseline to post-intervention (post minus pre) (16). The FMA-UE is a validated, reliable, and widely used scale for quantifying upper limb motor function after stroke.

Secondary outcomes included:

(a) Modified Barthel Index (MBI): used to assess activities of daily living (ADL). The MBI is a standard clinical measure for evaluating basic ADL performance and monitoring functional recovery before and after treatment, with well-established reliability.
(b) Mini-Mental State Examination (MMSE): used to evaluate global cognitive function. The MMSE is widely used internationally as a brief, standardized instrument to screen cognitive status and quantify the degree of cognitive impairment.
(c) Neuroimaging (fNIRS) outcomes.

### 2.7 fNIRS data acquisition

**Figure 5:**
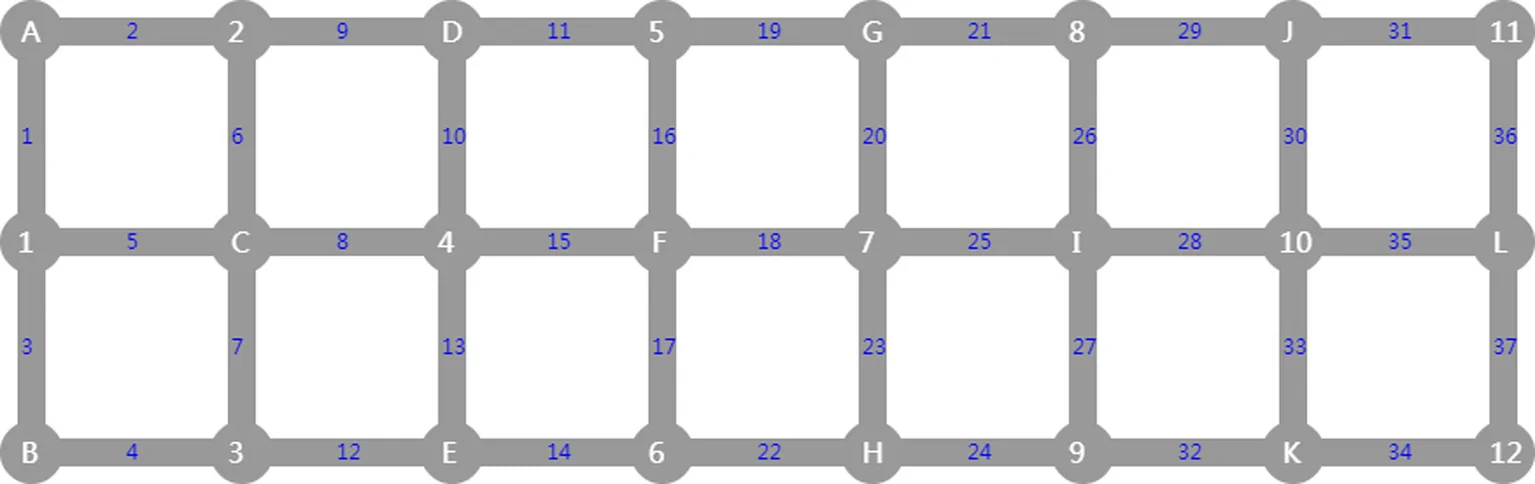
Schematic layout of the fNIRS probe/channel arrangement. Gray circles indicate optode positions on the fNIRS cap, and blue numbers indicate channel indices.

fNIRS data were acquired using a BS300 system (Wuhan Yiride Medical Equipment New Technology Co., Ltd., China). The device uses near-infrared light in the 650–900 nm range to detect hemodynamic changes approximately 2–3 cm beneath the scalp, reflecting changes in hemoglobin concentrations. The probe configuration comprised 12 emitters and 12 detectors, forming 37 measurement channels (Figure 5). The sampling rate was 10 Hz, and the system continuously recorded relative changes in local oxyhemoglobin (HbO), deoxyhemoglobin (HbR), and total hemoglobin (HbT) concentrations.

During acquisition, participants wore a cap-mounted optode array. Each source–detector pair constituted a channel that measured the mean hemoglobin concentration within the corresponding cortical region. The optode layout was positioned according to the international 10–20 system, with channel 19 located over Cz and channels symmetrically distributed over the left and right hemispheres (Table 1). The array predominantly covered the sensorimotor cortical areas. The cap was secured with straps around the head and under the chin to minimize motion.

**Table 1:** Anatomicl registration of channels.

Channel	MNI coordinates	Brodmann area (MRIcro)-brain region
	x y z	
CH01	−65 –9 36	PSC-L
CH02	−60 –20 50	PSC-L
CH03	−59 13 28	PreM & SMC-L
CH04	−51 27 34	DLPFC-L
CH05	−57 3 43	PreM & SMC-L
CH06	−52 –5 55	PreM & SMC-L
CH07	−48 19 48	DLPFC-L
CH08	−43 10 59	PreM & SMC-L
CH09	−44 –16 66	PSC-L
CH10	−33 –1 67	PreM & SMC-L
CH11	−24 –13 75	PreM & SMC-L
CH12	−39 30 49	DLPFC-L
CH13	−33 21 60	FEF-L
CH14	−19 32 59	FEF-L
CH15	−23 12 69	PreM & SMC-L
CH16	−14 –1 75	PreM & SMC-L
CH17	−13 22 67	FEF-L
CH18	−2 9 70	PreM & SMC
CH19	−4 –13 75	PreM & SMC
CH20	13 –1 76	PreM & SMC-R
CH21	22 –14 76	PreM & SMC-R
CH22	0 31 59	FEF
CH23	13 22 67	FEF-R
CH24	20 31 60	FEF-R
CH25	22 10 71	PreM & SMC-R
CH26	32–5 68	PreM & SMC-R
CH27	32 19 62	FEF-R
CH28	43 8 61	PreM & SMC-R
CH29	43 –17 69	PSC-R
CH30	52 –7 58	PreM & SMC-R
CH31	60 –23 53	PSC-R
CH32	40 28 52	DLPFC-R
CH33	49 17 51	DLPFC-R
CH34	53 26 37	DLPFC-R
CH35	59 3 46	PreM & SMC-R
CH36	66 –12 40	PSC-R
CH37	63 10 32	PreM & SMC-R

Resting-state acquisition. Resting-state fNIRS data were recorded for 10 min to obtain stable hemodynamic signals. Throughout the recording, participants were instructed to sit comfortably, keep their eyes open, maintain a stable head position, remain awake, and avoid intentional movements or excessive blinking.

**Figure 6:**
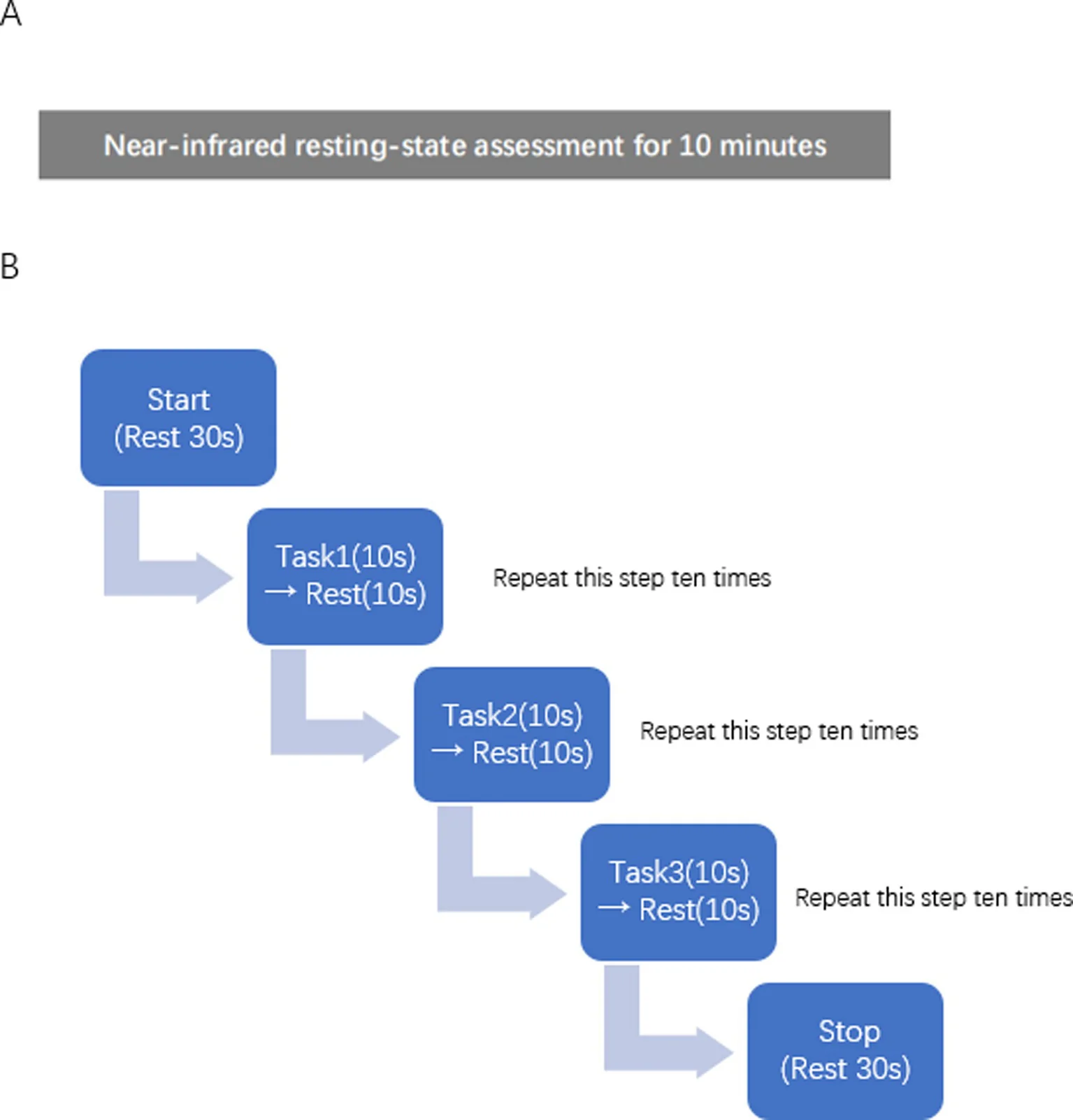
Schematic diagram of the fNIRS assessment paradigms. **(A)** Resting-state fNIRS assessment (10 min). **(B)** Task-state fNIRS paradigm. After an initial 30-s rest period, participants performed three motor tasks in sequence: Shoulder flexion (Task 1), Elbow flexion (Task 2), and Finger flexion (Task 3). For each task, a 10-s task period alternated with a 10-s rest period for 10 blocks, followed by a final 30-s rest period.

Task-state acquisition. For task-related recordings, participants sat upright and performed three motor tasks with the affected upper limb under the guidance: (a) shoulder flexion, (b) elbow flexion, and (c) finger flexion on the affected side. Each task was repeated 10 times. For each trial, participants were instructed to perform the movement for 10 s, followed by 10 s of rest. The total task recording time was approximately 10 min. The experimental paradigm for near-infrared measurement is illustrated in Figure 6.

The target movement was defined as an active movement performed within each participant’s available pain-free active range of motion while minimizing trunk displacement, shoulder elevation, and other compensatory movement patterns. Participants were instructed to perform each movement at a consistent self-selected pace following standardized verbal cues provided by the examiner throughout the recording.

To ensure standardized task execution, the examiner continuously monitored movement quality during all recordings. When compensatory movements were observed, verbal feedback was provided immediately to encourage correction. If necessary, minimal manual guidance was applied only before movement initiation to optimize limb positioning and was not used to facilitate or assist movement execution.

Because upper-limb motor impairment varied among participants, individuals who were unable to complete the full movement range were instructed to perform the maximal voluntary movement achievable within their active range of motion. Passive movement was not provided during fNIRS acquisition.

### 2.8 fNIRS data preprocessing

The fNIRS analyses focused on (i) task-evoked changes in oxygenated hemoglobin (HbO) (17) concentration under different motor paradigms and (ii) resting-state functional connectivity (FC) between predefined cortical regions of interest (ROIs) (18), in order to characterize cortical activation and network-level connectivity patterns within and between groups.

All preprocessing was performed in MATLAB R2014a (The MathWorks Inc., Natick, MA, USA) using the manufacturer’s data conversion utilities and the Homer2 toolbox, complemented by custom scripts. The main steps included data format conversion, visual inspection for data quality, artifact handling, and temporal filtering. To enable group-level comparisons referenced to a common lesion side, data from patients with right hemisphere lesions were flipped before statistical analysis, with the left hemisphere uniformly represented as the lesioned side.

#### 2.8.1 Resting-state fNIRS functional connectivity analysis

Raw light intensity signals were preprocessed in Homer2. Signals were converted to optical density and then to HbO concentration changes using the modified Beer–Lambert law. Motion artifacts were identified and corrected using the hmrMotionArtifactByChannel and hmrMotionCorrectionSpline algorithms from the HOMER2 package, followed by temporal filtering (low cutoff frequency 0.01 Hz, high cutoff frequency 0.1 Hz) to reduce slow drift and high-frequency physiological noise (19). All subsequent analyses in this study were based on HbO data, given their higher signal-to-noise ratio and sensitivity to state-related cortical hemodynamic changes in many fNIRS applications.

Functional connectivity (FC) (20, 21) was quantified at the region level. Channels were assigned to *a priori* cortical regions for visualization and summary analyses based on standardized optode registration, grouped into eight regions including bilateral DLPFC (Dorsolateral Prefrontal Cortex), FEF (Frontal Eye Field), PreM & SMC (Premotor and Supplementary Motor Cortex), and PSC (Primary Somatosensory Cortex) (22–24). ROI aggregation was performed by averaging the preprocessed HbO time series across all valid channels assigned to each ROI. For each participant, Pearson correlation coefficients were computed between the preprocessed HbO time series of every pair of regions. Correlation coefficients were Fisher z-transformed to improve normality. The resulting zFC matrices were used for group-level statistical inference.

#### 2.8.2 Task-related fNIRS preprocessing and activation analysis

For task data, raw files were first converted to the appropriate format, and then preprocessed to remove motion artifacts and physiological noise, as described above for resting-state data. HbO concentration changes were used as the primary indicator of cortical activation.

First-level GLM analysis. A general linear model (GLM) was implemented at the subject level. Task-related regressors were defined for each condition (shoulder flexion, elbow flexion, finger flexion), typically modeled with a boxcar function and convolved with an appropriate hemodynamic response function. GLM fitting yielded β-coefficients (β values) for each channel (25) or ROI and each condition, representing the amplitude of task-evoked hemodynamic responses.

ROI-level (26) effect estimation. For each participant, β values were averaged within predefined ROIs to obtain condition-specific activation estimates at the ROI level.

Group-level analysis. Group-level statistical analyses were then performed on the channel-wise β values. These analyses were used to evaluate intervention-related modulation of task-evoked cortical activation patterns and to compare activation responses between groups across time points.

### 2.9 Statistical analysis

All statistical analyses were performed using SPSS 25.0 (IBM Corp., Armonk, NY, USA) and MATLAB R2014a (The MathWorks Inc., Natick, MA, USA).

#### 2.9.1 Baseline characteristics

Continuous variables (e.g., age, disease duration, baseline FMA-UE, MBI, MMSE) were summarized as mean ± standard deviation (SD) or median (interquartile range) as appropriate; categorical variables (e.g., sex, stroke type, side of hemiparesis) were summarized as counts and percentages. Between-group differences at baseline (experimental vs. control) were examined using independent-samples t tests for continuous variables with approximately normal distributions, Mann–Whitney U tests for non-normal variables, and chi-square or Fisher’s exact tests for categorical variables.

#### 2.9.2 Behavioral outcomes

For FMA-UE, MBI, and MMSE, change scores were calculated as post-intervention minus baseline values. Within-group pre–post changes were examined using paired-samples t tests. Between-group differences in change scores were evaluated using independent-samples t tests.

#### 2.9.3 Resting-state fNIRS connectivity (zFC)

Resting-state functional connectivity was quantified as Fisher z-transformed connectivity values (zFC) between predefined region pairs. To assess within-group intervention effects, paired-samples t-tests were performed for each region pair by comparing post-intervention and pre-intervention zFC values separately within the experimental and control groups. To assess between-group differences at each time point, independent-samples t-tests were conducted for each region pair on pre-intervention data and post-intervention data, respectively. Multiple comparisons across region pairs were controlled using the false discovery rate (FDR) correction, and FDR-corrected *p* values < 0.05 were considered statistically significant.

#### 2.9.4 Task-related fNIRS activation

For task-state data, first-level general linear models produced β coefficients for HbO concentration changes in each region of interest and task condition (shoulder, elbow, and finger flexion). HbO β values were entered into a two-factor repeated-measures analysis of variance (ANOVA) with time (pre-intervention vs. post-intervention) as the within-subject factor and group (experimental vs. control) as the between-subject factor, to test the group × time interaction effect. A significant group × time interaction was interpreted as evidence that intervention-related changes differed between groups. For channels showing a significant interaction effect, *post-hoc* simple-effects analyses were performed. Multiple comparisons in *post-hoc* tests were corrected using the Bonferroni adjustment. Bonferroni-corrected *p* values < 0.05 were considered statistically significant.

## 3 Results

### 3.1 Results of baseline characteristics

Baseline demographic and clinical characteristics were comparable between the two groups. There were no significant differences between the two groups in terms of age (*p* = 0.402), gender (*p* = 1.000), hemiplegic side (*p* = 0.508), disease duration (*p* = 0.506), and stroke type (*p* = 1.000; Table 2).

**Table 2:** Comparison of baseline data between the two patient groups.

	Control group (*n* = 19)	Experimental group (*n* = 19)	*p-*value	*z-*value
Gender (Male/Female, n)	13/6	13/6	1	
Age [years, median (IQR)]	70 (65, 71)	67 (62, 72)	0.402	−0.865
Duration of illness [months, median (IQR)]	2 (1, 5)	1 (1, 5)	0.506	−0.687
Stroke type (Ischemic/Hemorrhagic, n)	16/3	15/4	1	
Side of dysfunction (Left/Right, n)	10/9	13/6	0.508	

### 3.2 Results of behavioral outcomes

Before treatment, there was no significant difference in FMA-UE scores between the two groups (*p* = 0.223). After treatment, the FMA-UE score of the control group improved slightly, but the difference was not significant, while the FMA-UE score of the experimental group showed significant improvement (*p* = 0.025). The improvement in FMA-UE was significantly greater in the experimental group than in the control group (Median between-group difference = 3.000 points, 95% CI 1.000 to 6.000; *p* = 0.012; see Table 3).

**Table 3:** Comparison of pre- and post-treatment FMA-UE scores between the two groups.

Variable	Control group (*n* = 19)	Experimental group (*n* = 19)	*p-*value	*z-*value
Before treatment [median (IQR)]	21 (11.31)	11 (7.27)	0.223	−1.229
After treatment [median (IQR)]	28 (13.38)	19 (15.41)	0.840	−0.219
Difference before and after treatment [median (IQR)]	6 (2.9)	9 (7.12)	0.012	−2.491
*p-*value	0.234	0.025^*^		
*z-*value	−1.198	−2.237		

*p < 0.05.

Before treatment, there was no significant difference in MBI scores between the two groups (*p* = 0.435). After treatment, the MBI score of the control group showed some improvement, but the change was not substantial, whereas the experimental group demonstrated significant improvement in MBI scores (*p* = 0.037). The Median between-group difference in MBI change was 0.000 points (95% CI −14.000 to 9.000; *p* = 0.506; see Table 4).

**Table 4:** Comparison of pre- and post-treatment MBI scores between the two groups.

Variable	Control group (*n* = 19)	Experimental group (*n* = 19)	*p-*value	*z-*value
Before treatment [median (IQR)]	54 (39.70)	50 (34.64)	0.435	−0.803
After treatment [median (IQR)]	61 (59.78)	61 (51.74)	0.506	−0.672
*p-*value	0.085	0.037^*^		
*z-*value	−1.724	−2.089		

*p < 0.05.

Before treatment, there was no significant difference in MMSE scores between the two groups (*p* = 0.234). After treatment, both groups showed significant improvement in MMSE scores (control group *p* = 0.025, experimental group *p* = 0.046). However, no significant between-group difference in MMSE change was observed (Median difference = −4.000 points; 95% CI −5.000 to 1.000; *p* = 0.525; Table 5).

**Table 5:** Comparison of pre- and post-treatment MMSE scores between the two groups.

Variable	Control group (*n* = 19)	Experimental group (*n* = 19)	*p-*value	*z-*value
Before treatment [median (IQR)]	25 (21.27)	20 (14.27)	0.234	−1.201
After treatment [median (IQR)]	28 (25.30)	24 (18.29)	0.525	−0.654
*p-*value	0.025^*^	0.046^*^		
*z-*value	−2.237	−2.000		

*p < 0.05.

### 3.3 Results of resting-state zFC

**Figure 7:**
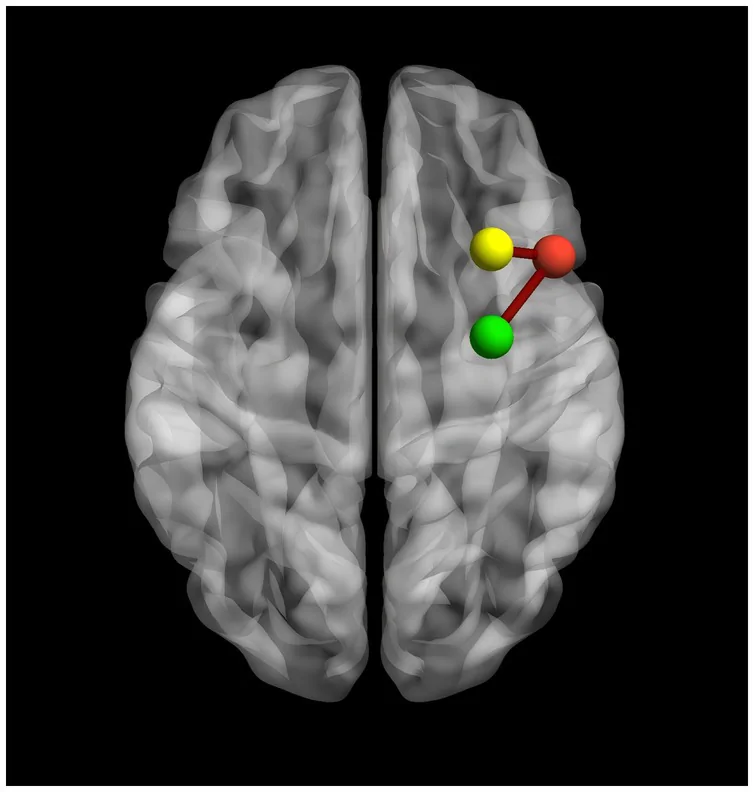
Schematic illustration of enhanced resting-state functional connectivity (zFC) in the experimental group after intervention (post > pre), based on fNIRS analysis. Enhanced connections were observed between PreM&SMC-R and DLPFC-R, and between DLPFC-R and FEF-R. Yellow, FEF-R; red, DLPFC-R; green, PreM&SMC-R.

Paired-samples t-tests were performed on the resting-state FC matrices (post-intervention minus pre-intervention) across eight regions of interest in each group. After FDR correction, no significant within-group changes were observed in the control group. In contrast, the experimental group showed significantly increased zFC after the intervention between PreM&SMC-R and DLPFC-R (t = 2.8939, *p* = 0.00967, Cohen’s dz. = 0.664), and between DLPFC-R and FEF-R (t = 2.8801, *p* = 0.00996, Cohen’s dz. = 0.661; Figure 7).

**Figure 8:**
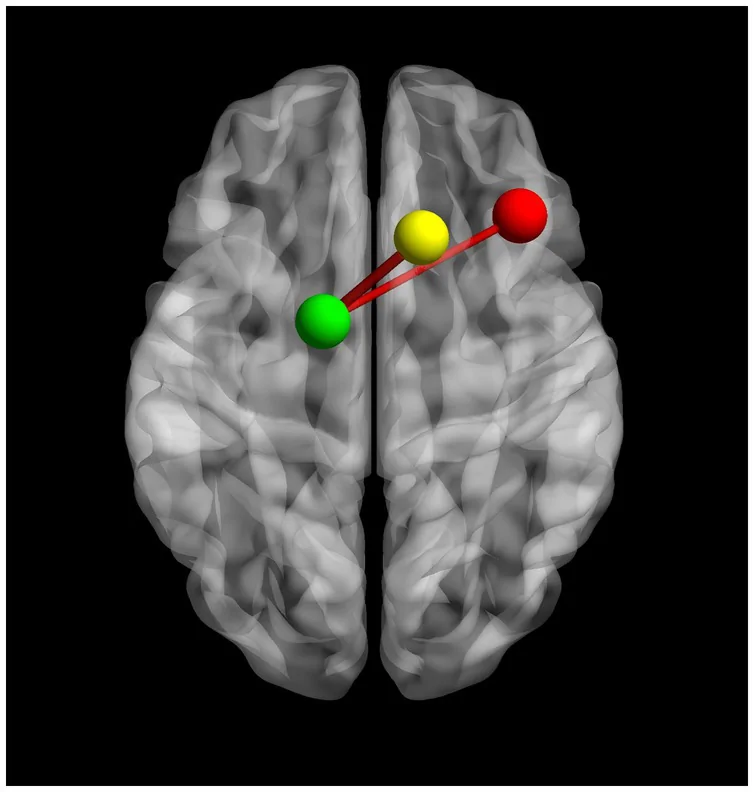
Schematic illustration of resting-state functional connectivity (zFC) differences between groups after intervention, based on fNIRS analysis. The experimental group exhibited significantly greater zFC than the control group between PreM&SMC-L and DLPFC-R, and between PreM&SMC-L and FEF-R. Yellow, FEF-R; red, DLPFC-R; green, PreM&SMC-L.

Independent-samples t-tests were then conducted to compare the two groups at each time point. After FDR correction, no significant between-group differences were found at baseline (pre-intervention). However, after the intervention, the experimental group exhibited significantly greater zFC than the control group between PreM&SMC-L and DLPFC-R (t = 2.4237, *p* = 0.02051, Cohen’s d = 0.786), and between PreM&SMC-L and FEF-R (t = 3.0643, *p* = 0.00411, Cohen’s d = 0.994; Figure 8).

### 3.4 Results of task-state HbO concentration

**Figure 9:**
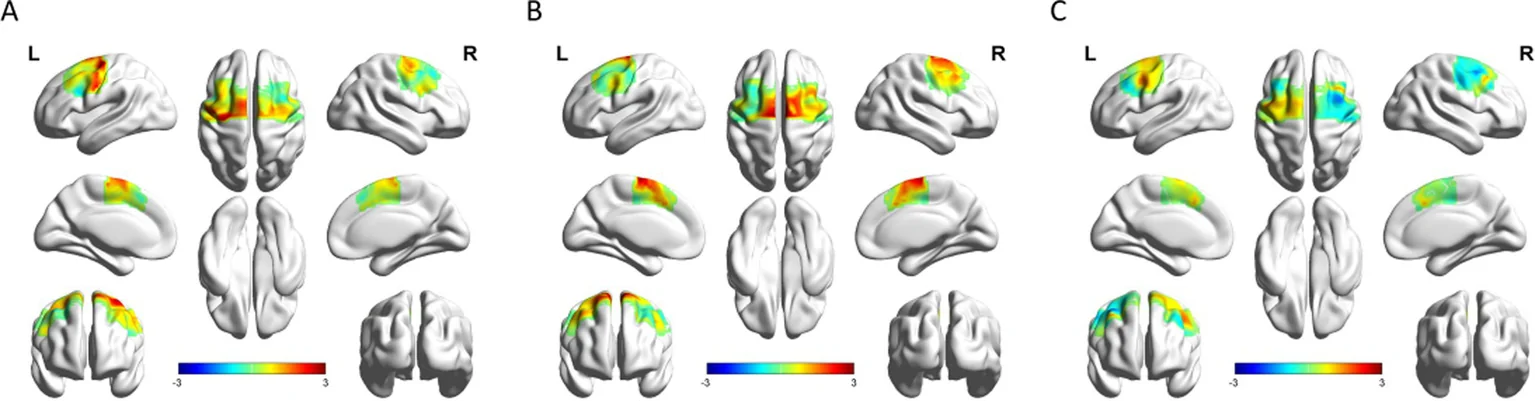
Schematic maps of task-evoked cortical activation measured by fNIRS in the control group before intervention. Shoulder flexion **(A)**; Elbow flexion **(B)**; Finger flexion **(C)**.

**Figure 10:**
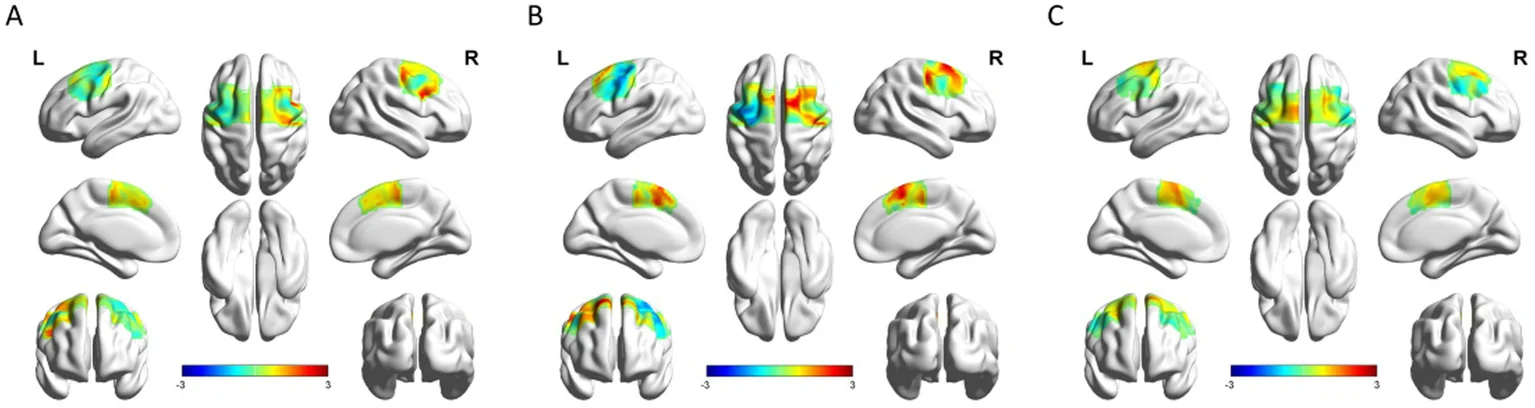
Schematic maps of task-evoked cortical activation measured by fNIRS in the experimental group before intervention. Shoulder flexion **(A)**; **(B)** Elbow flexion; **(C)** Finger flexion.

Task-based fNIRS cortical activation results showed that before intervention, both groups exhibited significantly increased activation in the ipsilesional and contralesional hemispheres during the three motor tasks, predominantly localized in the premotor and supplementary motor cortex (PreM&SMC; Figures 9, 10).

**Figure 11:**
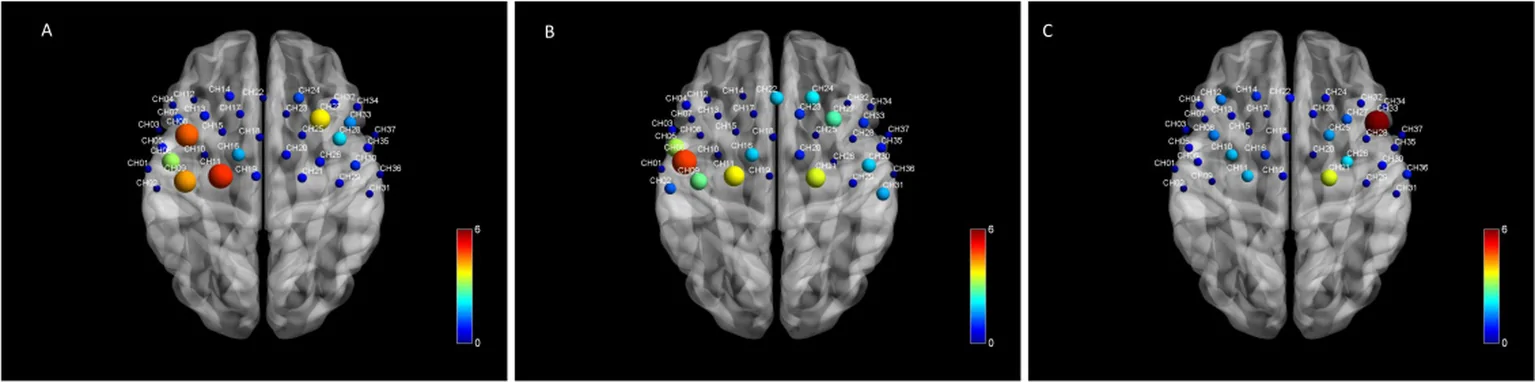
Schematic maps of group × time (experimental vs. control × pre- vs. post-intervention) interaction effects on task-evoked HbO cortical activation measured by fNIRS. **(A)** shoulder flexion; **(B)** elbow flexion; **(C)** finger flexion. The size and color of the nodes represent the magnitude of the *F*-value from the interaction effect (blue to red indicating values from 0 to 6; a larger *F*-value corresponds to a redder node color and a larger node size).

For the shoulder flexion task, significant group × time interaction effects were observed in the β values of CH8 (Channel 8, *F* = 4.668, *p* = 0.037, partial η^2^ = 0.115), CH9 (*F* = 4.325, *p* = 0.045, partial η^2^ = 0.107) and CH11 (*F* = 4.985, *p* = 0.032, partial η^2^ = 0.122). Bonferroni-corrected simple-effects analyses showed that cortical activation in the control group significantly decreased after the intervention at CH8 (M ± SE = −2.127 ± 0.904, 95% CI −3.962 to −0.293, *p* = 0.024). Similarly, a significant post-intervention reduction in cortical activation was observed in the control group at CH11 (M ± SE = −1.948 ± 0.775, 95% CI −3.519 to −0.377, *p* = 0.016). No significant simple effect was detected at CH9 after correction (Figure 11A).

For the elbow flexion task, the β values of HbO showed a significant interaction effect in CH6 (*F* = 4.846, *p* = 0.034, partial η^2^ = 0.119). However, Bonferroni-corrected simple-effects analyses did not reveal any significant differences at this channel (Figure 11B).

For the finger flexion task, HbO β values at CH33 showed a significant interaction effect (*F* = 6.415, *p* = 0.016, partial η^2^ = 0.151). Bonferroni-corrected simple-effects analyses indicated that cortical activation in the control group significantly decreased after the intervention at CH33 (M ± SE = -1.984 ± 0.763; 95% CI -3.531 to −0.437; *p* = 0.013; Figure 11C).

## 4 Discussion

In this randomized controlled trial, adding multimodal sensory-feedback robot-assisted training to dose-matched conventional rehabilitation resulted in greater short-term improvement in upper-limb motor function after stroke (27), as measured by FMA-UE. Improvements in activities of daily living (MBI) also favored the experimental group, whereas cognitive gains assessed by MMSE were observed in both groups without a clear between-group difference. Importantly, fNIRS findings suggested that these behavioral changes were accompanied by modulation of cortical hemodynamics and resting-state functional connectivity. Specifically, the experimental group showed enhanced resting-state connectivity within fronto-motor networks and task-related HbO changes in channels over motor- and prefrontal-related regions. Collectively, these findings support the interpretation that multimodal sensory-feedback robotic training may facilitate recovery by engaging both sensorimotor and higher-order control networks.

Previous fNIRS studies also have clearly captured a typical pattern: when patients attempt to move the affected upper limb, the activation of the primary motor cortex (M1) in the affected hemisphere is weakened, while the contralateral hemisphere exhibits overactivation. This phenomenon is considered a compensatory mechanism of the intact hemisphere. Mihara et al. found that under robot-assisted repetitive task-oriented training, this abnormal pattern dynamically changes. As training progresses and function improves, the activation level of the affected M1 area gradually increases, while the overactivation in the contralateral hemisphere tends to normalize. This suggests that effective rehabilitation training promotes brain functional reorganization, shifting from reliance on compensatory activation of the intact hemisphere to the reconstruction of the affected hemisphere’s network (17). In the present study, the experimental group demonstrated increased resting-state functional connectivity involving PreM&SMC and DLPFC/FEF after intervention, which is compatible with the notion that feedback-enriched robotic training modulates distributed cortical networks relevant to motor planning, control, and performance monitoring. However, these results should be interpreted cautiously, as the present design does not permit causal inference regarding the directionality of network changes.

### 4.1 Comparison with previous robot-assisted and multimodal rehabilitation studies

Our findings are broadly consistent with prior evidence indicating that robot-assisted upper-limb rehabilitation can enhance motor recovery after stroke by enabling high-intensity, repetitive, and task-oriented practice. Huo et al. found that under assisted mode, the extensive cortical response related to robot-assisted therapy observed in patients with severe dysfunction may contribute to brain functional reorganization during motor execution, which is considered the neural substrate of motor-related processes (28). A key strength of the present study is the dose-matched design, which minimizes the confounding influence of therapy time and supports the interpretation that the observed benefits are attributable to the qualitative features of multimodal sensory feedback rather than increased training duration alone.

Compared with conventional robot-assisted approaches that primarily emphasize trajectory guidance or passive assistance, the intervention used in this study integrated visual, auditory, and task-contingent performance feedback within a gamified training environment. Such multimodal feedback is thought to enhance motivation, attention, and error-based learning, thereby facilitating experience-dependent plasticity (29). The greater gains in FMA-UE and MBI observed in the experimental group suggest that enriching robot-assisted training with multimodal sensory feedback may improve the transfer of practiced movements to functional task performance in daily life.

### 4.2 Neurophysiological findings revealed by fNIRS

Beyond clinical outcomes, this study provides fNIRS-based neurophysiological evidence that multimodal robot-assisted training is associated with modulation of both cortical activation and functional connectivity. At rest, we observed strengthened connectivity in the experimental group within fronto-motor network pairs (including PreM&SMC-, DLPFC-, and FEF-related connections) after FDR correction. This pattern is consistent with the view that effective rehabilitation may promote re-integration of distributed cortical regions involved in motor planning, sensorimotor coordination, and top-down control.

During motor-task performance, task-evoked HbO analyses identified channel-specific group × time interaction effects across shoulder flexion, elbow flexion, and finger flexion tasks. Importantly, the channel-level simple-effects analyses in the present study did not show a significant post-intervention increase in task-evoked cortical activation in the experimental group; instead, significant changes were primarily observed in the control group, which showed reduced activation after intervention at selected channels. Previous fNIRS studies on stroke rehabilitation have shown that robot-assisted training can significantly enhance activation in the primary motor cortex and supplementary motor area, which may contribute to the recovery of motor planning ability in stroke patients (30, 31). Therefore, the task-state fNIRS findings are better interpreted as a between-group difference in the direction and stability of cortical response modulation, with the experimental group showing a relatively preserved activation profile rather than a generalized activation increase.

The involvement of prefrontal regions (e.g., DLPFC-related channels/connectivity) is noteworthy. In feedback-rich rehabilitation settings, DLPFC recruitment may reflect enhanced attentional allocation, action monitoring, working memory demands, and goal-directed control during motor execution (32). Thus, the present results support a broader network-level account in which multimodal robotic training engages not only canonical motor regions but also executive-control systems that may contribute to learning and performance optimization.

### 4.3 Potential mechanisms linking multimodal feedback to motor recovery

The observed behavioral and neurophysiological effects can be interpreted within a framework of enhanced sensorimotor integration. Multimodal sensory feedback provides continuous information about movement accuracy, timing, and success, which may strengthen the coupling between sensory input and motor output (16, 17). Such feedback-driven learning is known to engage fronto-motor networks and to promote adaptive reweighting of neural resources after stroke. The concurrent enhancement of resting-state connectivity and task-evoked activation observed in this study is consistent with a model in which repeated, feedback-enriched practice induces both state-dependent and task-specific plasticity, ultimately supporting functional recovery of the paretic upper limb (3).

### 4.4 Clinical implications

From a clinical perspective, the present findings suggest that multimodal sensory-feedback robot-assisted training can be feasibly integrated into conventional rehabilitation programs without increasing overall therapy time, while providing additional motor benefits. The use of fNIRS further highlights the potential value of portable neuroimaging tools for monitoring cortical responses to rehabilitation and for informing the development of mechanism-based, individualized training strategies in stroke recovery.

Lesion laterality and baseline motor impairment are both recognized sources of inter-individual variability in post-stroke recovery. Although these factors were balanced between groups through randomization and therefore were unlikely to confound the primary between-group comparisons, the present study was not powered to determine whether either variable modified treatment responsiveness. Future adequately powered trials should evaluate lesion laterality and baseline impairment as potential moderators of both behavioral recovery and cortical reorganization.

## 5 Limitations

Several limitations of this study should be acknowledged. First, the sample size was relatively modest and the trial was conducted at a single center, which may limit the generalizability of the findings. Larger, multicenter studies are needed to confirm the robustness of the observed effects across diverse patient populations. Second, the intervention and follow-up period were relatively short, and only immediate post-intervention outcomes were assessed. Consequently, the durability of the behavioral improvements and neurophysiological changes remains unclear. Future studies incorporating longer intervention periods and extended follow-up assessments are warranted. Third, fNIRS measures hemodynamic responses from superficial cortical regions and cannot capture activity in deeper brain structures, such as the basal ganglia or thalamus, which are also critically involved in motor recovery after stroke. In addition, HbO changes represent an indirect proxy of neural activity and may be influenced by systemic physiological factors despite careful preprocessing. Besides, although parallel improvements in behavior and cortical measures were observed, causal relationships between changes in functional connectivity, cortical activation, and motor recovery cannot be established in the present design. Future studies incorporating brain–behavior correlation or mediation analyses may help to clarify these relationships. Finally, although lesion laterality and baseline motor impairment were balanced between groups through randomization, the present study was not powered to determine whether either factor modified treatment responsiveness. Future multicenter studies with larger sample sizes should evaluate these clinically important variables using prespecified interaction analyses.

## 6 Conclusion

This randomized controlled trial indicates that multimodal sensory-feedback robot-assisted training, when added to dose-matched conventional rehabilitation, may provide superior short-term improvement in upper-limb motor function after stroke. These functional gains are accompanied by modulation of cortical activation and resting-state functional connectivity measured by fNIRS, suggesting engagement of distributed sensorimotor and executive-control networks. Together, the findings support the potential of multimodal robotic training combined with portable neurophysiological monitoring as a promising strategy for mechanism-informed, individualized stroke rehabilitation.

## Data Availability

The original contributions presented in the study are included in the article/supplementary material, further inquiries can be directed to the corresponding authors.
